# Report of a Series of Experiments on the alleged Mental Sympathy of a persion said to be in a state of Mesmeric Somniloquism

**Published:** 1844-03

**Authors:** 


### WESTERN JOURNAL

OF

### MEDICINE AND SURGERY.

MARCH, 1844.

### Art. I.—*Report of a Series of Experiments on the alleged Mental Sympathy of a person said to be in a state of Mesmeric Somniloquism.* By an Association of Gentlemen.

EDITORAL NOTICE.

The experiments recorded in the following Report were made at our suggestion, on a young lady lately exhibited publicly in this city. The interest excited by these public exhibitions, here and elsewhere, may justify us in publishing an account of the series of private experiments of which we give the entire record. Several of them it would have been desirable to repeat, but opportunity for doing so did not occur. The Report after being drawn up was submitted to the gentlemen who made the experiments, who decided, unanimously, that it presented the facts of the case correctly. Indeed, they were copied with very great care from the original records. After discussing the report, it was ordered to be published in this Journal, but as some of the gentlemen wished their names withheld, the whole have been suppressed. Had their names gone with it, their high respectability for intelligence and integrity, would have given additional assurance of the truth of what the report contains. While preparing the report, we were led to draw up a speculative paper on the Philosophy of Mesmeric Somniloquism, which was read immediately after that document, and will appear in our next number. D.

### ANALYTICAL REPORT.

The Committee to whom was referred the record of the experiments lately made by themselves and their colleagues, on Mesmerism, with instructions to transcribe, analyse and present in a digested form, the facts presented in it, have sought, faithfully, to accomplish the same; and respectfully submit to the scrutiny and consideration of their associates, the following REPORT:

### MODE AND OBJECT OF THE EXPERIMENTS.

The object proposed by the association, was to ascertain the truth of the assertion that the individual who has been brought by the manipulations of Mesmerism into a somnambulic and somniloquent state, is susceptible of the thoughts and feelings of the person who may be in communication, independent of all known modes of intercourse. To this end it was agreed in writing, that each member of the association, should describe the objects or scenes which he intended to present to the somniloquist, not making them known to any person whatever, and sealing the paper hand it to the member appointed to keep the same; that he would put no leading question to the somniloquist; that her answers should be listened to and reported, by members of the association, written down by others, sealed up, and delivered to another member than the one who held the other sealed papers, and that, at the close, the two sets of papers should be opened and compared; all which has been observed.

### OF THE MESMERIZATION.

The somnambulic and somniloquent state was, at all the meetings of the association, produced in the same manner, in about the same time and presented the same phenomena. Seated in front of the young lady, her mesmeriser, pressed the balls of his thumbs against hers and looking steadily in her face, it immediately assumed a subdued and quiet aspect. She did not look into his eyes, but directed her own a little downwards. Very soon a slight dilatation of the pupils occurred, to which succeded a little quivering of the eye-balls, and shortly after they rolled upwards and outwards, the lids instantly falling, and remaining down throughout the whole course of the experiments. The time occupied by the mesmerization was from two to four minutes. In three out of the four meetings, the state of the pulse before and immediately after that process was observed and recorded, and was as follows: First, before, 82 in a minute, after, 96; second, 80 before, 88 after; third, 82 before, 96 after. In two of the meetings, the pulse was examined towards the close, and found to have fallen to its natural standard. Her respiration did not appear to be changed, nor was there any sensible change in the heat of her hands and face. No spasms or cataleptic rigidity of her muscles was observed, nor any difficulty of articulation. The experiments of each afternoon, lasted about an hour and a half, during which she sat erect and unsupported on a sofa; and when brought out of the somniloquent state, seemed not to be fatigued, and declared that she was neither tired nor indisposed. No experiments were made to ascertain the reality of that state, as the mode of inquiry adopted, did not require a previous decission of that point. Without affirming, the association admitted the reality of her somniloquent condition.

SCENES AND OBJECTS RECOLLECTED BY THE GENTLEMEN IN COMMUNICATION.

As many of these were of a complex character, the committee have found it necessary to an intelligent view of the conformity of her answers, to analyse them, and present the whole in a numerical series, to which they have referred for the respective topics in writing out her answers.

*Topics of Mr. A. B.*

1. A gold watch with a white dial-plate, held in the hand.

2. The room in which the experiments were made.

3. Experiments on Mesmerism in a different room, all the persons present, being three gentlemen and one lady.

4. The interior of the Unitarian church, Louisville; as a public Mesmeric lecture room, two gentlemen in conversation with a mesmerized lady.

*Of Mr. C. D.*

5. The cave house, near the Mammoth Cave, with a view of its colonade in front.

6. Enter the cave, preceded by a guide carrying torches; light up a part with Bengal light; pass through a narrow passage to the river; cross it, and enter Cleaveland's cabinet.

7. Enter a row-boat on the (Ohio) river, clad in a hunting dress carrying a gun; proceed to six-mile island; and return against a strong wind, creating large waves with white caps.

8. Shooting wood-cook on the island.

9. The room in which the experiments were made, and the persons therein.

10. A silver lancet case, opened and the lancets exposed.

*Of Mr. E. F.*

11. The steamer Buffalo partially aground in the straits, St. Mary, near Lake Superior. The rocking of her by the passengers in large numbers running from side to side, on the upper deck; the engines in motion at the same time; the green shores on each side; a white awning over part of the deck; a group of ladies with a French mesmerizer in their midst.

12. Infant baptismal ceremony in Christ's Church, Cincinnati; the pastor alone within the chancel; an assemblage of infants with their nurses and parents in front; considerable number of spectators; some of the children crying.

13. The last scene repeated.

14. The same, again repeated.

15. Solitary walk, on a bright day, upon the outer side of Santa Rosa Island, in the Gulf of Mexico; white drifted sands on each side and behind; scattered ever-greens; trees and shrubs; wave after wave breaking in white foam on the sand beach; two or three ships at a distance.

16. The same scene reproduced.

*Of Mr. G. H.*

17. The steeple of St. Paul's Church in this city.

18. My office, table and books.

19. The amphi-theatre of the Institute, filled with pupils, and a recent subject on the table.

20. The Washington Monument in Baltimore; a view of the city and surrounding country.

21. Public mesmeric lecture in the Unitarian church, filled by the audience; a gentleman in conversation with a mesmerized lady; (same as No. 4).

22. Professor C. in his study, arm chair, books and writing implements.

23. The Falls of Niagara.

24. A human skull.

25. Dr. B.'s person looked at.

*Of Mr. I. J.*

26. A Hebrew bible, held in the hand.

27. A "scene" in the House of Representatives at Washington, at night; a storm without and great confusion in the hall; the persons of Mr. Adams, Mr. Stanley, and Governor Pope.

28. The room in which the experiments are made to-day; the description of the room; persons present; how many; who? &c. (Same as No. 9.)

*Of Mr. K. L.*

29. Ancient church (England); large stone edifice, supported by heavy buttresses; windows arched; square tower; the great western door of remarkable architectural beauty.

30. An old yew tree.

31. Castle close by; front view; a round tower in ruins; a series of remains containing dungeons, &c.; a long low wall extending to a high tower; John of Gaunt's gateway.

32. Bull-baiting; bull fastened with a long rope to a ring set in a large stone; large concourse of people; a man sets a dog on the bull; the bull tosses him in the air; another dog set on and pins the bull.

33. Conical glass furnace; the making of glass.

34. Glass cutting.

*Of Mr. M. N.*

35. Mammoth Cave Hotel; company of ladies and gentlemen at supper-table.

36. After supper they visit the cave, the guides bearing lights; they enter; stop to drink at the spring; they proceed along the high archway; look at the cottages for invalids; proceed to the river which they can't cross, the boat being lost; on their return diverge to take a view of *Gorin's dome brilliantly lighted.* (Same as No. 6.)

37. Visit to Harrodsburg Springs; arrival; see company promenading on long piazza and on lot; Dr. Graham advances to receive us; we stand on the steps and look at cottages.

38. Then visit the spring through a vista of small trees; looking at pond on right hand.

39. Find gentlemen and ladies at saloon, and receive glass of water from waiter at fountain.

40. Amphi-theatre of Institute; enter by door of students and take a seat fronting Dr. C. lecturing; look at skeletons on right and left; and the Venus and Apollo beyond. (Same as No. 19.)

*Of Mr. O. P.*

41. A rolling mill in operation, in which I will bring forward the steam engine with its great fly-wheel in motion; the powerful cast iron rolls in full operation, drawing the red-hot bar; the nail machines also in operation.

42. I will imagine myself on board a steam boat approaching one of the locks and dams on the Kentucky river; also whilst in the lock, and the appearance from the deck on opening of the flood-gates, and passing out into the river above.

43. I will present my residence from Walnut street.

44. An interior view, as presented in the hall and parlour.

*Of Mr. Q. R.*

45. Harrodsburg Springs; the cottages right and left; trees; old hall with its porch; people in it. (Same as No. 37.)

46. Road to the spring; pond on the right. (Same as No. 38.)

47. Water trickling down at the springs; the house over them. (Same as No. 39.)

48. Residence of Col. Thompson; the house; yard; apartments; the family circle; fire; a young lady and gentlemen at a table; the library.

49. Green-house.

50. The anatomical theatre; skeleton; Venus; Apollo; Mannikin; the Professor; the class. (Same as Nos. 19 and 40.)

CLASSIFICATION OF HER ANSWERS.

To facilitate the study of her answers, the committee, in transcribing the whole, have thrown them into two great classes. First, those in which they had more or less conformity to the topics which occupied the attention of the gentlemen in communication with her; second, those in which her answers were almost negative, or, being positive, were, on the whole, foreign from the subjects of thought at the time. The former class, they have subdivided into cases of the first, second, and third degree of conformity, the last embracing the lowest instances of relevant answers. Thus the whole are presented in a regular series, commencing with the strongest and descending to the weakest conformity, until they end in the false and negative.

### CLASS I.

*Answers in the First degree of Conformity.*

*No. 27.—Mr. I. J.*

Q. Where are we now? A. Seem to be in a room. Q. Large or small? A. Large. Q. Day or night? A. I should think it was night. Q. Is the room lighted? A. Yes, on one side. Q. What do you see in the room? A. There seem to be persons in the room. Q. Are there many persons? A. Not a great many. Q. What do they seem to be doing? A. Seem to be still, doing nothing, don't see any ladies there. Q. Hear any noise? A. I hear a noise that sounds like water. Q. Look on one of the gentlemen, and tell how he looks. A. He seems to be an old gentlemen, standing. Q. Do you see that gentleman over there? A. He don't seem to be so large as the other, not quite so tall. His hair goes back over his head, his eyes seem to be shut. Q. Hear any noise? A. Noise of persons talking. Q. How are the lights arranged? A. They seem to be arranged on the sides and one large light before us. Q. What else do you see? A. That gentleman seems to be moving his face, don't look very pleasant sometimes; seems to be something white on one arm. Q. Look again and see if you see anything? A. I only see one arm. Q. Is the other arm hid behind his chair? A. He don't seem to have but one arm.

*No.* 5.—*Mr. C. D.*

Q. What do you see? A. A house, a large house. Q. What peculiarity? A. Columns in front. Q. Many of them? A. A good many, high ones.

*No.* 38.—*Mr. M. N.*

Q. Where are we now? A. A little elevated, looking off. Q. What do you see? A. Something like water there. Q. Where are we going now? A. Seem up. Q. How are we travelling? A. Should think walking. Q. Is it along a street? A. Not. Q. What objects do you see? A. Trees, large dark things.

*No.* 17.—*Mr. G. H.*

We'll take a very short walk. Q. Are you fond of looking at pretty things? A. Sometimes. Q. What am I looking at? A. A building. Q. What appearance, high or low? A. High. Q. How is the top? A. High, goes to a point. Q. How many rows of windows? A. Three all round it.

*Answers in the Second degree of Conformity.*

*No.* 29.—*Mr. K. L.*

Q. Do you love to travel? A. Yes. Q. Would you like to take a voyage to England? A. Yes. Well! we'll go as both agree. Q. See anything now? A. A building. Q. Large or small? A. Large, high, comes up round on the top; large building, top not in a point. Q. What sort of windows? A. Large, round, large door, one part dark, the light of the door. Q. How many rows of windows? A. Two. Q. What built of? A. Looks like stone, don't know. Q. Look old or new? A. Looks old, round in front.

*No.* 1.—*Mr. A. B.*

Q. Do you see anything now? A. You seem to have something in your hand. Q. What is it? A. I don't see it distinctly. Well! look closely at it, and then describe it to me. A. It looks like a watch. Q. What sort? A. A gold watch. Q. What face; gold or white? A. White. Q. What do you see on its face? A. Don't see anything. Q. Look close at it, see if you can see the hands. A. No sir, don't see the hands. Q. Would you like to see the hands if you could? A. Yes sir. I see one hand. Q. Can you see what it points to? A. It points to the figure three. Q. Can you see the other? Are you still trying to distinguish it? No, sir, don't see the other one. Q. Do you see anything else? A. I do not see anything but the watch.

*No.* 42.—*Mr. O. P.*

I'll present you an interesting scene: I'm showing you a great deal at once. A. Don't seem to be in a room, but standing up on something high and light colored. Q. We'll look off. What is before us? A. Looks like persons. Q. Look again at its extended scenery. A. Seems to be moving along like persons. Q. What else do you see? A. Something like water. Q. Is it calm or disturbed? A. Moving on one side, still on the other. Q. A great deal of it? A. Great deal. Q. Does it look like a lake or pond or ocean or what? A. Like a fall on one side, other see nothing. Q. See water fall very distinctly? A. Not very. Q. Why call it a fall? A. The water seems to come down. Q. Take your eyes off and look in front and see if you can describe the things. A. Something bright like fire. Q. See if there be any changes; now what do you see? A. Seems to be a dark appearance. Q. See any more distinctly now? A. See persons standing near the bright. Q. We will go further and you must speak of what is now before. Do you perceive anything? A. Something very high, not distinctly. Q. Begin at the foundation low down. A. Dark, an open place there. Q. What looks dark? A. The object. Q. What is it, rock, wood, or mud? A. More like a rock. Q. Going further up, what do you see then. A. Something large, and looks brighter. Q. Describe; is it buildings, trees, or what. A. Hills. Q. Anything on them? A. Some light, some dark.

*Answers in the Third degree of Conformity.*

*No. 20.—Mr. G. H.*

Q. Will you cross the Alleghany mountains with me? A. Yes. Q. What do you see now? A. A building. Q. Describe it! A. Light colored. Q. Tell all about it! A. Large building, something in front like columns. Q. Is it high or low? A. Not very high nor very low. Q. What do you see as we look from the top? A. Something like water one side. Q. Anything else? A. Something like a hill, high. Q. Anything else? A. Good many buildings, another large building.

*No. 32.—Mr. K. L.*

Q. We'll take something else. Like something to amuse you, won't you? A. Yes. Q. Will you walk to my father's house? A. Yes. Q. What do you see there? A. Water. Q. Anything else? A. Something moving. Q. What does it look like? A. Persons, looks dark, moving, number of persons. Q. See anything among them? A. Something high, of different colors, tall person. Q. See any beasts? A. Dark, looks like some kind of animal. Q. See what the beast is doing! A. Walking, with something over its head, large head. Q. Are they doing anything to it? A. Putting something over its head that is red. Q. What do you call it? A. Never saw such an animal before. Q. Does it stand still? A. No. Q. Are people looking at it? A. Some people above, looking at it, seem to be upon it. Q. See any more than one animal? A. No sir. Q. Is it pleasing or offensive? A. Looks very well.

*No.* 12.—*Mr. E. F.*

Q. I see something now that looks light enough to be seen distinctly: I wish you to look at it closely. A. It looks very much like a building: Seems to be dark on one side of us, and light on the other side. Q. Well, what do you see besides? A. I see persons. Q. Are you able to tell what they are doing? A. Persons seem to be standing. Q. Are they standing still or walking about? A. Some seem to be walking about. Q. Can you tell what they are doing? A. Seem standing about something, cannot tell what it is. Seems to be dark. Q. Does it appear to be day or night? A. Day. Q. What else do you see but persons? A. Something above us. Q. What does it look like? A. Seems to be bright. Q. Can you tell me any thing about it? A. No sir, I don't see it distinctly. Q. Can you see any thing else? A. No sir, I don't see any thing else very distinctly, I don't see any thing else there. Q. Can you distinguish among these people any one different from the rest? A. No sir, nothing distinct from the rest; I see something white around them, but dont see any thing else. Q. Do you see any thing else now? A. No sir.

*No.* 43.—*Mr. O. P.*

Q. Feel as if you were seeing well for me this evening? A. Tolerable. Could'nt see at all for the gentleman before. Q. I'll give you another object—see any thing? Something moving. Q. Tell all about it. A. See something like a house. Q. What about it like a house? A. See something like a door —open like a door—not a very pretty looking house—something on top. Q. Large or small house? A. Large. Q. What colour? A. Can't tell, not white, not red, kind of dark colour. Q. Is it as long as it is high? A. Longer. Q. Would you like to enter it? A. Yes. Q. How can we get in? A. Have to go in at a small place by going round.

*No. 2.—Mr. I. J.*

Q. Do you see any thing now? A. See a building. Q. Have I any thing in my hand? A. I see something white. Q. Any other colour? A. I see something black. Q. Any other colour? A. Something red. Q. Look at it good now. A. It looks like a book. Q. Think it's a book? A. Looks like it

### CLASS II.

### FALSE OR NEGATIVE ANSWERS.

*No. 2—Mr. A. B.*

Q. Well now look closely—Do you see any thing? A. I see something that looks bright. Q. Bright like what? A. I don't know what it is, looks bright. Q. Do you see any person? A. Yes sir. Q. Do you know the person? A. I've seen the person before, I dont know him very well. Q. Are you still looking at that person? Think you know who that person is? A. Dont know that person's name. Q. Lady or gentleman? A. A gentleman. Q. Do you see any other person? A. Yes sir, I see another person. Q. Do you know that person? A. No sir. Q. What sort of place do you seem to be in? A. A room. Q. Do you see any other person but the one? A. I see a lady. Q. Tell me who that lady is, can't you tell the lady's name? Q. Do you see her dress? A. Dark colour. Q. Do you see the lady more distinctly than you did? A. Yes sir. Q. Think now that you know her? A. I think I know her. Q. Think you know her name? A. No sir. Q. Do you see her distinctly enough to be sure you dont know her name? A. Yes sir. Q. Do you see any other person besides that lady? A. I see a gentleman. Q. Do you see him distinctly? A. No sir, not distinctly. Q. Do you see him more distinctly than before? A. Seems to be behind something, cant see him distinctly. Q. Do you see any thing else there? A. No sir. Q. Do you see any other people there? A. No. sir. Q. Will you go to another place? A. If you wish to go.

*No.* 3.—*Same gentleman.*

Q. Do you see any thing now? A. Seem to be standing on something. Q. Please tell me if you see any thing or person there! A. I see a person. Q. Do you see the person distinctly. A. I see a gentleman. Q. Did you ever see that gentleman before? A. Yes sir. Q. Do you think you know him? A. No sir. Q. What does he seem to be doing? A. I don't see that he is doing any thing. Q. Do you see any other person? A. No sir. Q. Look closely and see if you can? A. No sir. Q. Do you see the same gentleman? A. Yes sir. Q. Sitting or standing? A. Standing. Q. Do you see any thing else? A. I see something that seems to be on the Wall. Q. I'll look at the object, and if you can see any thing tell me. A. I see something that seems to be moving. Q. Do you see any thing else? A. I see something that looks like a painting. Q. Do you see that person that you saw a while ago? A. No sir. Q. Do you see any one? A. I see a gentleman. Q. Do you know him? A. No sir. Q. Do you see him distinctly? A. No sir.

*No.* 4.—*Same gentleman.*

Q. Do you see any thing before your mind now? A. I see something like water. Q. Please to look closely at it, and tell me if you see any thing else? A. I see something like a room there. Q. Any body in it? A. There's a person in there. Q. What does the person seem to be doing? A. There seems to be something the matter with the person. Q. A gentleman or lady? A. A gentleman. Q. Look closely at him, and see what he's doing. A. I don't know what he's doing. Q. Do you know him? A. I do not. Q. Do you observe any other object. A. No sir.

*No.* 6.—*Mr. C. D.*

Q. What do you see? A. In a room. Q. A large one? A. Yes, a number of persons. Q. Sitting or standing? A. Sitting; one now standing. Q. Are we still or walking? A.

Still. Q. Is it light or dark? A. Light in back part more than in front. Q. What do you see? A. Person seems to be laying down. Q. Is the room dark? A. Dark at that end. Q. What appearance now? A. I don't see anything; dark. Q. How is it now? A. It seems to be light. Q. See anything but a room? A. Not in a room now. Q. See anything? A. Seem to stand on something bright. Q. Quite high? A. Yes. Q. Standing still or going? A. Not in a room; seem to be walking. Q. Now see anything? A. Something off there like water. Q. Are we near it? A. Yes.

*Nos.* 7 *and* 8.—*Same Gentleman.*

Q. Is my dress changed any? A. Long dark dress. Q. What do you see? A. Something like a light. Q. Where are we? A. Seem to go under some long place. Q. Anybody with us? A. Yes, but looks dark. Q. What are they doing? A. Kneeling down. Q. Look again; any difference? A. No sir. Q. Let's return. What way are we returning to Louisville? A. By land, not water. Q. What conveyance? A. In something goes fast. Q. How does the road seem. A. Small, narrow, dark on sides.

*No.* 9.—*Same Gentleman.*

Q. Returned—where are we now? A. In a room. Q. Large or small? A. Large. Q. Any persons? A. Yes. Q. Many? A. Don't know. Q. Still or moving? A. Moving and talking. Q. See any person you ever saw before? A. Yes, the gentleman who was talking with me just now. Q. Any ladies? A. No.

*No.* 10—*Same Gentleman.*

Q. Anything in my hand? A. Yes, a book. Q. What kind? A. A dark one. Q. Large or small? A. Small. Q. Its color? A. Dark. Q. Any ladies in the room? A. No ladies, only gentlemen.

*No.* 11.—*Mr. E. F.*

Q. Are you tired of sitting up? A. No sir. Q. How long since you came to Louisville? A. I don't know. Q. Travelled about it much? (No reply). Q. Do you prefer the city or the country? A. If the person likes it best I do. Q. How do you like the scene before us now? A. Don't see anything before me? Q. How do you like the appearance now? A. It seems to be dark around us. I do not see anything distinctly. Q. Which of those things before us now do you think most beautiful? A. I don't see anything at all. It appears to be dark. It don't seem to be in a room. Q. Can you distinguish nothing? A. No sir. Q. What seems to be the reason? A. I don't know sir. Q. Is it not light enough for you to see the difference between land and water? A. I can't see distinctly. Q. Does not that look like water? A. It doesn't look like water.

*No.* 13.—*Same Gentleman; No.* 12 *repeated.*

Q. Do you observe anything now? A. No sir. Q. Can you tell where we are? A. We seem to be in a room. Q. What do you see? A. Nothing; my mind seems to be dark; there is nothing before it. Q. Do you see no person in the room? A. No sir. Q. Do you see no furniture? A. No sir. Q. Well! look attentively and see if you can't discover something. Q. What kind of a room does it seem to be? A. A very long one. Q. Do you see nothing in it? A. Yes sir; a person. Q. What does he seem to be doing? A. He seems to be sitting down. Q. Do you see nothing else in the room but the person? A. There are other things in the room, but I don't see them distinctly. Q. Well! examine them carefully. Do you see no article at all? A. I see something in the room that seems to be large and dark. I can't see it plainly. Q. Do you see anything on the walls? A. I don't see the walls. Q. Why do you think it is a room then? A. It seems to be more like a room than anything else. Q. Do you see anything like a carpet? A. I don't know what you mean by a carpet. The floor seems to be hard. Q. Do you see any chairs? A. No sir. Q. Do you see any stove or fire place? A. No sir. Don't see anything that looks like fire in the room. Q. Do you see more than one person yet? A. No sir, I see only one person.

*No.* 14.—*Same Gentleman; being a repetition of the last.*

Knows the gentleman. Q. How do you like the appearance of what we are looking at now? A. We seem to be out of doors. Q. What do you see before us now? A. It seems to be very high. Q. What does it seem to be? A. I don't see it distinctly; there seem to be trees there. Q. What do you see now? A. I see something moving. Q. What do you see now? A. Something before us that seems attached to something—different colors on it. Q. What do you see now, anything different from the last? A. No sir; I see that same thing. Q. What do you see now? A. I see persons. Q. What do you see now? A. I see something that looks like water.

*No.* 15.—*Same Gentleman.*

Q. What do you see now? A. I don't see anything now. Q. Do you see anything now? Do you not see any part of it now? A. No sir. Q. Nor now? A. No sir. I can't see anything. Q. Do you observe anything now? A. I see something of different colors; I don't know what it is. Q. What do you see now? A. I don't see anything at all. It seems to be dark. Q. Do you hear anything? A. No sir. Q. Do you see anything now? A. No sir, I don't see anything at all. Q. I think you can't fail to see whether it is night or day, the thing now before my mind. A. I see nothing before my mind. Q. Well! what do you see now? A. I don't see anything. Q. What seems to be the reason that you don't see anything now? A. I don't know sir.

*No.* 16.—*Same Gentleman; the last repeated.*

Q. Do you observe anything now? A. No sir. Q. What do you observe now? A. I don't see anything at all. Q. Do you see anything now? A. No sir.

*No.* 18.—*Mr. G. H.*

Let's go somewhere else. Q. What do you see now? A. Looking into a room. Q. What do you see? A. Something like a painting. Q. What are you now looking at? A. Something; one side looking like a figure. Q. What sort? A. A

person. Q. What am I now looking at in the room? A. Something dark. Q. More than one? A. Yes, many. Q. Anything like them before? A. One looks like a head.

*No.* 19.—*Same Gentleman.*

Let us get out of this little room; I'm tired. Q. What looking at now? A. In a room. Q. Is it larger than the other? A. A different kind of room. Q. How arranged? A. Nothing in the centre; many around. Q. Describe them. A. Don't see anything distinctly. Q. Look again. A. See a number. Q. What like? A. Lower parts white; seem to be round; nothing like them before. Q. Look again in this big room? A. Persons; a great many. Q. Young or old? A. Young and some old. Q. Any ladies present? A. See only two. Q. What at this moment? A. Something dark going round. Q. Anything on what is going round? A. Don't know. Q. Describe it. A. Don't see it distinctly. Something bright projecting from the side. Q. What is on the thing moving round? A. Can't see it distinctly.

*No.* 21.—*Same Gentleman.*

Let us get back over the mountains. Q. What do you see now? A. Seem to be in a room, large. Q. Ever here before? A. Yes. Q. See a number of things? A. Yes. Q. What is most interesting? A. Don't see anything—something like a painting. Q. What else? A. No particular object. Q. Anything now? A. A person standing by something. Q. Gentleman or lady? A. Gentleman. Q. What is he standing by? A. Hand resting on something, light and bright at sides. Q. What are the persons doing? A. Sitting down. Q. What are they doing? A. Can't see. Q. Getting tired of this little room? A. It's not very small. Q. Any more persons? A. See but one. Q. Ever been here before? A. No; I thought I had, but think I have not.

*No.* 22.—*Same Gentleman.*

Q. Let us walk a little. A. Yes. Q. What do you see? A. Nothing. Q. Try again. A. See nothing at all. Q. Tired of me, are you not? A. Seen so much. Q. What do you see? A. Some persons in a room, light, some noise. Q. What is most prominent? A. Something standing in the room, with something over it. Q. Look again, something worthy of your attention. A. Don't know what.

*No. 24.—Same Gentleman.*

Q. What am I looking at? A. Something like a house. Q. What is it? A. Don't see it distinctly; seems high. Q. Look again; what like? A. Don't know; don't see it distinctly. Q. Have you seen it before? A. Yes—like it? Q. Look again. A. Don't know.

*No. 25.—Same Gentleman.*

Let us get off from that object. Q. Are you tired? A. Yes, of looking. Q. I'll take you but to one object more, and not far off. Q. What am I looking at? A. Trying to think. Q. Look again. A. Seem to be standing in a small place. Q. What object? A. Something moving. Q. Look again. A. Stationary now. Q. What sort of object? A. Large and of different colors. Q. Ever see it before? A. No.

*No. 28.—Mr. I. J.*

Q. Now where are we? A. Out of doors. Q. What do you see? A. A building there. Q. Tell me what you see, what sort of a building? A. Don't see it distinctly. Q. see any people? A. A number of people seem to be standing upon something.

*Nos. 30 and 31.—Mr. K. L.*

We'll turn our backs and look at something else. Q. Now what do you see? A. Some persons there. Q. What does it look like, a building, or what? A. Open on the sides. Q. Well, we'll walk a little further. What do you see? A. Something like trees. Q. Any thing above the trees? A. Something never saw before; looks bright. Q. What to the left hand? A. A low building. Q. What to the right? A. Something like water. Q. To the extreme right? A. Looks dark. Q. Does it look large or small? A. Large. Q. Look like a building? A. Does not, never saw any thing like it.

Large, dark, open in front, level on top, looks smaller on top, but don't know. Q. See any hill or plain? A. A hill to the right.

*No.* 34.—*Same Gentleman.*

Well! we'll go into another part of the building. Q. What do you see? A. Something dark moving round. Q. See any persons? A. Seem to be standing, hold something in their hands. Q. How are they dressed? A. Light colored dresses. Q. Anything on their heads? A. Something dark, long, turns down. Q. What in their hands? A. Can't see distinctly, something colored. Q. How do you like the jaunt to England—a pleasant voyage? A. Yes. Q. Suppose we return to the United States. A. Yes.

*No.* 35.—*Mr. M. N.*

Let us make an excursion and view some objects. Q. What are we approaching, what do you see? A. Some persons. Q. Where are we now? A. Seem to be standing on something now. Q. What do you see around? A. Seems to be a great many things, nothing distinctly.

*No.* 36.—*Same Gentlemen.*

Let us move a little further. Q. Where are we now? A. Seem to be in a room. Q. We're still moving, where are we now? A. Seem standing, now, before something. Q. What do you particularly observe? A. Something seems to be hanging up. Q. Where are we now? A. Go so fast I can't say. Q. What do you see now? A. Don't see distinctly, looks bright to me. Q. Where are we now? A. Seems to be a figure there of something, we are standing. Q. What do you see now? A. Don't see anything distinctly.

*No.* 37.—*Same Gentleman.*

Q. You're tired of this excursion, are you not? A. No, but I can't see distinctly. Q. What do you see now? A. A building. Q. What sort? A. A dark colored. Q. Is any thing coming towards us? A. Yes, something dark.

*No.* 39.—*Same Gentleman.*

Q. Where are we now? A. Standing in a small place, number of things round us. Q. What do you particularly remark, what's that in the middle of the place? A. Seems to be round. Q. What does it look like? A. Don't know what, something, a kind of animal. Q. What is that doing, in the middle of the place where we are? A. Seems to be moving round.

*No.* 40.—*Same Gentleman.*

We'll go to another place which will interest you very much. Q. What do you see before you? A. Seem to be in a large place, something round before us. Q. What is it? A. Something like a building, don't think it is. Q. Where are we now? A. Looks like a room now. Q. Where are we now? A. Seem to be standing in some place. Q. What is right before you there? A. Don't know, don't see it distinctly. Q. Look farther! A. Seems to be high. Q. A little beyond, a few steps, what do you see? A. Seems to be moving down.

*No.* 41.—*Mr. O. P.*

Q. In what direction are we moving? A. Can't tell. Q. Are we going up or down? A. Up. Q. What is the surrounding scenery? Tell us what you see in looking round? A. See something that seems to be moving. Q. What sort of motion? A. I can't describe, seems to go up. Q. Is it all in one direction? A. sometimes a little down and then moves off. Q. Is there any other object? A. Something seems to be moving round. Q. Any interest about it? A. Moving round with something bright about it. Q. Look beyond and describe! A. Something like a house. Q. I want to bring an object of a clear and distinct kind, try to see it. A. Something light colored, seems very pretty, rather large, not very high, something in the centre. Q. Describe that in the centre! A. Seems to be rather tall, comes out rounding on the sides, singular, never saw it before. Q. Any peculiar color? A. Two or three, blue, more like that than any other.

Q. Are we in a house or not? A. Not in a house. Q. How is it? A. Something over us, not a house. Q. Well, we'll change to another part, tell me what you see, see anything? A. See different colors, and something at the side of us like a dog. Q. Look just before you, rather look low than high, and describe the object. A. Seems to be long. Q. Do you see any persons? A. One person, standing on that thing. Q. Moving or stationary? A. Still, and something in his hand, seems to hold it out left side. Q. Anything on the right? A. Something high. Q. Any men about them? A. Three. Q. Using their arms? A. One hanging down, the other hand raising it up.

*No.* 44.—*Same Gentleman.*

Let us go in and first examine the floor. A. It is hard, not like wood. Q. What like? A. Stone. Q. What color? A. Brown. Q. All of one color or more? A. Color that looks white. Q. Tell on the right hand wall what? A. Something hanging up like figures. Q. What on the left? A. Goes in and looks open. Let us enter the right-hand apartment, and then look at the floor and tell me what it looks like? A. Seems to be white. Q. Cast your eye all over and describe the room; what is there worth speaking of? A. Something on the side that sticks out, left-hand side, something bright on the right-hand side that hangs down. Q. I'm fixing my mind on a particular matter, do you see anything distinctly? A. Something before us of different colors. Q. Tell us the colors. A. Seems white, and like a red and a dark, a dark color, not exactly red. Q. What does it look like? What intended for? A. Don't know what it is. Q. We'll go further on, we've now another object, tell me what it is? A. Seems to be a figure of something. Q. Is it a male or a female? A. A female. Q. When you speak of a figure, do you mean a painting or a piece of statuary? A. A statue. Q. Now another object, do you perceive it? A. Not distinctly. Q. See it any better? A. Something over the top, looks like a painting. Q. Look smooth or rough? A. Rough. We'll go further and see another object, do you see it? A. Yes.

Q. What is it like? A. I do not understand. Q. Are you tired? A. No, but don't see distinctly. Q. Something before me quite interesting, do you perceive it? A. Seem to be in a room now.

*No.* 45.—*Mr. Q. R.*

Q. Do you see anything before you? A. Don't seem to be in a room. Q. Do you see anything before or around you? A. Something high, but not a building. Q. What seems before you now? A. See some person.

*No.* 46.—*Same Gentleman.*

Q. Let us walk further; see anything now? What does it seem to be? A. Can't see distinctly. Q. Appear to be water? A. See water there. Q. Anything about it? A. That side something above the water of different colors?

*No.* 47.—*Same Gentleman.*

We have a pleasant walk before us now. Q. Do you see anything pleasant? A. Going into a small place. Dark there in the front part. Q. See anything there? A. Gone into a small place, dark; seem to be looking at something. Q. Can you describe it? A. Seems to be something round. Q. Can you see what is in it? A. Dark, round sides, seems to be bright.

*Nos.* 48 *and* 49.—*Same Gentleman.*

Q. We'll walk again; a new scene. A. Yes. Q. We have a pleasant prospect now; do you see anything? A. Something like water; see a building. Q. What's its appearance? A. Rather singular form, something goes round. Q. Do you see the water now? A. See water. Q. What do you see? A. Something there; think they are persons—two. Q. Where are we now? A. Like a room, long. Q. What do you see? A. Something bright above, going down before. Q. What now? A. Don't see that distinctly. Should think we are in a room. Q. Can't you see? Look if there be anything before us? A. Something long like a table, high, raised up; something white over it. Q. Do you hear anything? A.

Like striking and talking. Q. What kind of noise? A. Loud. Q. See anything now? A. Something like a man. Q. See anything? A. Something that side of the room seems to be red. Q. What does it look like now? A. Seems to be long. Q. We'll change and go out of this room; see anything before us now? A. No sir, don't see it distinctly. Something seems to be hanging up there. Q. See something now? A. See something white there going across; see persons there. Q. What are they doing. A. Seem to be standing; now stooping down.

*No. 50.—Same Gentleman.*

We shall not be able to walk long this evening; must change now and go to another scene: Q. See anything now? A. Seem to be going up—walk. Q. Have you ever been here before? A. Yes, I think I have; seem to be still now. Q. What strikes you now? A. Something very high; seems to be noise there; moving; building there the other side. Q. See anything striking beiore you now? A. Something; looks very singular. Q. What does it look like now? A. Seems to be something painted. Q. See more than one before you? A. Yes, three not alike. Q. What have you before you now? (No answer). Q. Same as before. A. Not distinctly; something seems to be standing up. Q. What does it look like? A. Figure of something. Q. What? A. Like a person; top part seems large; something else about it; seems to be something round it. Q. Do you like to look at it? A. Is pleasant; something over the top that don't look pleasant. Q. What is your eye directed to now? A. Should think it a person. Q. Appear to be moving or still? A. Standing still; sometimes moving. Q. See it distinctly? A. See the person distinctly; something over the head of it. Q. See anything else? A. No sir. Q. Look now and see if you recognize anything. A. Don't seem to be in a room. Q. Where do we seem to be? A. Seem to be standing in a place; something over us. Q. What seems before us? A. Seems dark. Q. Seem to be moving? A. Moving; dark. Q, See anything now? A.

Something on the side of it, bright, dark round it. Q. See anything distinctly now? A. No sir, nothing now.

### MANNER, LANGUAGE AND STYLE OF HER ANSWERS.

The record of questions and answers submitted to the committee has been carefully examined, and the first remark they have to make upon it, is that the somniloquist seems to have been silent except when she was addressed, as they no where find a single observation made by her, but in reply to a question, or in consequence of a remark made by the gentleman in conversation with her. This taciturnity shows itself still further, in the brevity of her answers, which were generally limited to the enunciation of a single idea, or conception of the mind, which in every instance, except two or three, where a leading question was put in relation to sound, involved visual perceptions only. In comparing or uniting her answers on the same topic, it is obvious, that she seldom had a clear conception of it; and they scarcely find any evidence of her perceiving the relations of the different members of any group. Nearly one hundred of her replies were entirely negative; in quite as many more, she saw something, but too indistinctly to give any account of it; in a great many cases, her enunciation of an object was prefaced by the expressions, "looks like;" "resembles;" "seems like;" "appears as if;" and "seems to be;" when she answered in a more confident manner, the reply does not in general extend beyond a single quality or characteristic of the object, such as color, form, position or motion, often imperfectly or partially expressed and scarcely ever referred to an absolute standard. Thus her vocabulary is barren, and her style eminently vague and obscure. In a great number of cases her answers, of course, are susceptible of various applications and interpretations; and it is obvious to the committee, that in many instances, she herself did not make an application of the qualities or characteristics which she announced to any particular object. The committee have not been able to discover any connexion between the distinctness of her answers and their truth, for some of the most obscure and even negative, relate to the objects which were really present in the minds of the gentlemen conversing with her, or actually before their eyes; while some of the most explicit, refer to other objects than those which occupied their attention. The catalogue of subjects exhibited in the analysis just given, must be admitted to present a great variety, but there is by no means a corresponding variety in her answers. On the contrary, they are abundant in set phrases. Among these the committee find, and may cite—"the figure of something;" "a small place;" "something moving;" "something moving round;" "something that looks dark;" "something white;" "something painted," or of "different colors;" "something that looks bright;" the last of which the committee have counted fourteen or fifteen times, while many of the others recur eight or ten times. "A person" or "persons" occurs about thirty times, generally when they were present, but did not make more impressive parts of the group than things; a "house," "building," or "room," occurs twenty-three or twenty-four times, seventeen of which, it should be stated, correctly, the remainder erroneously; and in a few instances she made no allusion to either, when it should have been done. She announced water four times when it was present, four times when it was absent, and failed to indicate it four times, when it made a part of the landscape. Once she saw it after a leading question, twice failed to see it under a leading question, and once under a leading question she heard it. In all cases, except one or two, she was confused and uncertain as to its appearance and extent; which, however, was the case with almost every other object. Her answers generally presented something which could be applied to familiar scenes and objects, but not to those of a remote and uncommon kind, in which houses, furniture, and persons were not the principal matters. Hence she said nothing which was applicable to the boat-scene on the Lakes, the excursion on the Ohio, the walk on Santa Rosa Island, or the descents into the Mammoth Cave.

### REPETITION OF THE SAME SCENES.

This accidentally occurred with several of them. Thus, No. 19 was presented by three different gentlemen; Nos. 37, 38 and 39 by two; No. 4 by two; No. 9 by two; No. 12 three times by the same gentleman, and No. 15 twice by the same; affording an opportunity of comparing her accounts to different gentlemen, or to the same at different times, of no less than eight scenes. A reference to the numerical catalogue will afford an index to these cases, the study of which is vitally connected with our inquiry; for while identity of answers, on the same scenes, to different gentlemen, would establish the reality of their mental influence on the somniloquist, diversity, would of course, go to prove it an illusion. The committee have carefully compared these answers, but do not feel it their duty to state the conclusions to which they have come.

### MODE AND EFFECT OF QUESTIONING.

Although the members of the association had subscribed a promise, not to put leading questions, until the influence of others had been exhausted, still the committee have discovered that most of the members, no doubt unintentionally and unconsciously, did put questions, calculated, not merely to elicit an expression of the state of mind of the somniloquist, but to modify it. Thus, when she answered—"a watch," "a book" or "a building," she was questioned concerning its form, size, color and other characteristics, and of course gave some account of it, which was as full when that was not the object which the gentleman had sought, mentally, to present to her, as when it was. But it was especially in reference to change of object, or locality, that this mode of interrogation occurred. Thus, no less than forty questions or remarks appear in the record, which could not fail to suggest such changes.

The committee wish to refrain from all speculations on the influence of such interrogatories and remarks; but they may be allowed to state as a fact established by the record itself, that in the cases in which replies of the most positive kind, whether true or false, were given, this mode of questioning was most apparent; while answers of a negative character were oftenest returned to questions, which could suggest nothing to the mind of the somniloquist or those around her.

All which is respectfully submitted.

DAN. DRAKE,
L. P. YANDELL,
Committee.

Louisville, February 8, 1844.

